# Vaccinations in Pediatric Hematology and Oncology: Biologic Basis, Clinical Applications, and Perspectives

**DOI:** 10.3390/vaccines13040397

**Published:** 2025-04-10

**Authors:** Baldassarre Martire, Alessandra Beni, Maria Felicia Mastrototaro, Veronica Santilli, Giorgio Ottaviano, Davide Montin, Caterina Rizzo, Mayla Sgrulletti, Michele Miraglia del Giudice, Giorgio Costagliola, Viviana Moschese

**Affiliations:** 1 Unità Operativa Complessa (UOC) of Pediatrics and Neonatology, Maternal-Infant Department, “Monsignor A.R. Dimiccoli” Hospital, 70051 Barletta, Italy; liciamastrototaro@gmail.com; 2 Department of Clinical and Experimental Medicine, University of Pisa, 56126 Pisa, Italy; alessandrabeni95@gmail.com; 3 Research Unit of Clinical Immunology and Vaccinology, Academic Department of Pediatrics (DPUO), IRCCS Bambino Gesù Children’s Hospital, 00165 Rome, Italy; veronica.santilli@opbg.net; 4 Department of Pediatrics, Fondazione IRCCS San Gerardo Dei Tintori, 20900 Monza, Italy; g.ottaviano88@gmail.com; 5 Division of Pediatric Immunology and Rheumatology, “Regina Margherita” Children Hospital, 10126 Turin, Italy; davide.montin@gmail.com; 6 Department of Translational Research and New Technologies in Medicine and Surgery, University of Pisa, 56126 Pisa, Italy; caterina.rizzo@unipi.it; 7 Pediatric Immunopathology and Allergology Unit, Policlinico Tor Vergata, University of Rome Tor Vergata, 00133 Rome, Italy; maylasg@gmail.com (M.S.); moschese@med.uniroma2.it (V.M.); 8 Department of Woman, Child and of General and Specialized Surgery, University of Campania “Luigi Vanvitelli”, 80138 Naples, Italy; michele.miragliadelgiudice@unicampania.it; 9 Section of Pediatric Hematology and Oncology, Azienda Ospedaliero Universitaria Pisana, 56100 Pisa, Italy; giorgio.costagliola@hotmail.com

**Keywords:** asplenia, chemotherapy, chimeric-antigen receptor T cells (CAR-T cells), hematopoietic stem cell transplantation (HSCT), immune reconstitution (IR), immunization, influenza, pneumococcus, meningococcus

## Abstract

Children with hemato-oncological diseases represent a heterogeneous population at heightened risk for vaccine-preventable diseases. Their immunosuppressed state reduces vaccine efficacy and raises safety concerns regarding live attenuated vaccines due to the risk of viral reactivation. The immunological and clinical implications of the single conditions are significantly different; therefore, specific vaccination strategies are needed. Despite the availability of vaccine guidelines for immunocompromised patients, clinical practice remains highly variable. It is generally recommended to avoid vaccinations during chemotherapy, with some exceptions for influenza, pneumococcal, and, in some countries, hepatitis B vaccines. The timing of immune recovery after chemotherapy depends on the specific treatment and most guidelines recommend administering vaccines 3–6 months after treatment cessation. Concerning HSCT, the timing of immune recovery is affected by several factors such as the HSCT platform, graft-versus-host disease (GvHD), and infections. Inactivated vaccines are typically administered 3–6 months post-HSCT, while live attenuated vaccines are delayed for at least two years. In children with asplenia or hyposplenism, recommendations focus on immunization against encapsulated bacteria, with tailored schedules based on the patient’s age and underlying condition. This paper explores the biological factors influencing vaccination efficacy and safety in pediatric hematology and oncology patients. It also provides an updated overview of the available evidence and current vaccination guidelines. Finally, this paper highlights the main clinical and research areas for further improvement to provide tailored vaccination schedules for this vulnerable population.

## 1. Introduction

The vaccination of children with hematologic and oncologic diseases is a significant challenge in clinical practice. Children with malignancies, those undergoing hematopoietic stem cell transplantation (HSCT), and those with hematologic conditions causing anatomical or functional asplenia (e.g., hemoglobinopathies), are at a substantially increased risk of vaccine-preventable diseases. At the same time, their immunosuppressed status raises critical concerns regarding both the efficacy and safety of vaccinations. Indeed, it is well-known that most of the specific aspects of the immune response to vaccines can be impaired in this category of patients. This specifically involves the germ center reactions (class switching, somatic hypermutation, and increase in antibody affinity), T and B-cell cooperation, and the adequate generation of immunological memory. Despite the availability of various guidelines, significant variability persists in their application across clinical settings. Moreover, in recent years, the advent of novel therapeutic approaches, such as monoclonal antibodies (mAbs), small molecule inhibitors, and chimeric antigen receptor (CAR)-T cell therapies, reshaped treatment paradigms. This introduced additional complexities to immunization strategies, as recent advancements created unique clinical scenarios and new immunization requirements. In this paper, we review the immunological implications of chemotherapy, HSCT, and asplenia and provide an overview of the research areas and the most updated guidelines for vaccinating children with hemato-oncological diseases.

## 2. Vaccinations in Children Receiving Chemotherapy

### 2.1. Rationale: The Burden of Immunosuppression and Immune Recovery

Chemotherapy is a cornerstone of pediatric oncology, offering life-saving treatments for a wide range of malignancies. However, its immunosuppressive effects increase susceptibility to infections, many of which could be prevented by routine vaccination. Immunosuppression during chemotherapy arises primarily from the myelosuppressive nature of the drugs, leading to neutropenia, lymphopenia, and the impaired production of functional immune cells [1]. The impact on immune function varies with the patient’s age, the type of cancer, and the intensity of the chemotherapy regimen [2]. In children, chemotherapy-induced immunosuppression is more pronounced due to their developing immune systems. Young children rely heavily on innate immunity, which is significantly compromised during chemotherapy. Furthermore, chemotherapy induces lymphocyte depletion, marginally affecting natural killer (NK) cells but significantly reducing circulating CD3+ T cells [3]. B cells also undergo profound depletion, often resulting in abnormally low immunoglobulin levels [4]. However, immunoglobulin levels tend to recover during the transition from intensive to maintenance therapy [2]. Regarding antigen-specific immunity, limited data exist on the gradual decline of vaccine-induced antibody titers during chemotherapy. However, studies consistently show that, by the end of treatment, many children exhibit antibody levels below protective thresholds [4], even if they had completed their vaccination schedules before starting chemotherapy [5,6,7,8]. Consequently, children with cancer are at an increased risk for both common infections (e.g., viral upper respiratory infections) and severe opportunistic infections caused by fungi and bacteria. These infections often exacerbate the underlying condition and delay further treatment, underscoring the critical need for a comprehensive vaccination strategy to reduce infectious complications.

### 2.2. An Overview of Current Guidelines: Vaccinating Patients During Chemotherapy

Vaccination in children undergoing chemotherapy is a complex issue that requires careful consideration of timing, vaccine type, and individual patient factors. Several international and national health organizations provide guidelines that help clinicians address the challenges of vaccinating this category of patients (Table 1). These include the Centers for Disease Control and Prevention (CDC) [9], the Infectious Diseases Society of America (IDSA) [10], and the Italian Association for Pediatric Hematology Oncology (AIEOP) [11].

#### 2.2.1. Vaccination During Chemotherapy

Routine vaccinations are generally deferred during active chemotherapy due to the diminished immune response and the high risk of vaccine inefficacy. Additionally, live vaccines are contraindicated due to the potential for vaccine-associated diseases [11]. For this reason, it is of paramount importance at this stage that the patient’s close contacts are vaccinated with live attenuated vaccines [12]. Exceptions include the annual inactivated influenza vaccine (IIV), pneumococcal vaccine, and COVID-19 vaccines, which may be administered under specific conditions. Despite the suboptimal immune response to influenza vaccination during chemotherapy, the risk of severe influenza infection justifies its administration during flu season. Additionally, studies show that influenza vaccination can elicit protective antibody responses in children undergoing chemotherapy. This is particularly relevant in less intensive treatment phases, such as maintenance therapy for acute lymphoblastic leukemia (ALL) [13,14]. However, the impact of vaccination on clinical outcomes and seasonal morbidity remains uncertain. Other non-live vaccines may be administered during maintenance therapy; notably, according to CDC guidelines, the doses are considered valid only if protective antibody titers are confirmed. In countries with high hepatitis B (HBV) prevalence [15,16], in cases where the HBV vaccine was not administered previously, it is recommended to vaccinate patients during chemotherapy. In this case, optimal timing is pivotal in order to maximize immune response and minimize interactions with the treatment. While vaccine responses may be weaker in seronegative patients undergoing chemotherapy, HBV vaccines remain safe and well-tolerated [15,16].

#### 2.2.2. Vaccination After Chemotherapy

Upon completion of chemotherapy, patients enter a phase of progressive immune recovery. This process is gradual, with the recovery timeline influenced by the malignancy type, chemotherapy regimen, and patient age [4]. Lymphocyte populations, including T and B cells, start to recover, with most patients achieving normal functional levels within six months. However, CD4+ T lymphocytes recover more slowly and may remain reduced in 20–50% of patients one year after treatment cessation [4]. Post-chemotherapy immunoglobulin levels also frequently decline, requiring up to a year to normalize. Overall, immunosuppression may persist for 6–12 months [1]. Inactivated vaccines can generally be reintroduced 3–6 months after chemotherapy, once the patient’s absolute lymphocyte count (ALC) has rebounded to at least 1.0 × 10^9^/L [5,10]. This timing ensures a more robust and durable immune response. For children whose vaccine schedules were interrupted at the start of chemotherapy, guidelines typically recommend resuming the program from the last suspended dose. In this case, the possibility of administering booster doses is considered [11]. The timing for assessing immune recovery after chemotherapy is not standardized, and the specific thresholds of immunoglobulin and lymphocyte subpopuluations for reintroducing vaccines are lacking. Similarly, there is no uniform agreement on the opportunity to evaluate specific antibody titers to guide the vaccination strategy. In clinical practice, it is reasonable to suggest performing an immunological assessment at the end of chemotherapy and 3–6 months after its cessation. This can be performed to reintroduce non-live vaccinations when ALC is recovered. There are no major immunological limitations for the development of an adequate response (i.e., severe hypogammaglobulinemia, severe CD19+ or CD4+ lymphocytopenia).

Recommendations for live attenuated vaccines, such as MMR and varicella, are more variable. The CDC suggests that these vaccines may be administered three months after chemotherapy. However, for regimens involving anti-B-cell antibodies, live vaccine administration should be delayed for at least six months, contingent upon evidence of sufficient T-cell recovery. Some disease-specific protocols recommend deferring even non-live vaccines for at least 12 months post-chemotherapy [17]. Therefore, clinical practice remains highly variable. The assessment of lymphocyte subpopulations could help in ruling out severe CD3+, CD4+, or CD8+ lymphocytopenia before the administration of live vaccines.

## 3. Beyond Conventional Chemotherapies: Vaccinating Children Receiving Monoclonal Antibodies and CAR-T Cell Therapy

### 3.1. Vaccinating Children After Treatment with New Drugs

In recent decades, a therapeutic revolution took place with the development of new therapies targeting cytokines and receptors involved in disease pathogenesis, such asmonoclonal antibodies (mAbs) and small molecule inhibitors (e.g., JAK inhibitors). These treatments represent a significant advancement in the management of hematological malignancies, providing more specific and less toxic options compared to conventional chemotherapy. However, the wide variety of biological mechanisms and immunological effects associated with these therapies limited the availability of studies on vaccine recommendations and immunogenicity in this patient population. Consequently, most vaccination guidelines are based on expert opinion [18,19,20]. Although no definitive evidence suggests an increased risk of pathogen reactivation in patients treated with biological therapies or JAK inhibitors, live attenuated vaccines remain contraindicated. If necessary, live vaccines should be administered at least 4 weeks before starting treatment (6 weeks for alemtuzumab) or postponed until therapy has ceased. The waiting period for vaccination after the end of treatment varies depending on the specific drug (Table 2). On the other hand, inactivated vaccines are considered safe, though their efficacy may be reduced by drug-induced immunosuppression. Some studies indicate that patients on biologics may exhibit immune responses to inactivated vaccines comparable to or better than those in control groups [21,22]. Notably, these data derive from single observational studies and explore only the effects of some specific biologics. Therefore, to assess the likelihood of responding to a vaccination and individualize the vaccination schedule, clinicians should consider several factors. These include the patient’s clinical history, comorbidities, treatment regimen, risk group status, and the epidemiological context.

As a general rule, it is important to highlight that, when therapy can be planned, non-live vaccines should also be administered at least 4 weeks before treatment initiation to optimize immune responses. On the other hand, for therapies that cannot be delayed, vaccination should be scheduled a few days before the next drug administration. This will allow ALC to optimize at the time of vaccine administration and, therefore, the response to vaccination.

In patients undergoing biological or JAK inhibitor treatments, some vaccinations are of specific relevance. These include IIV during flu season and sequential pneumococcal vaccines. Moreover, the administration of the HBV vaccine and hepatitis A vaccine in cases of drug-induced hepatotoxicity (e.g., blinatumomab, inotuzumab, alemtuzumab) is strongly suggested when feasible [19].

Special consideration is required for drugs with potent immunosuppressive effects on B-cells, such as anti-CD20 antibodies (i.e., rituximab) and anti-CD19 antibodies (i.e., blinatumomab). Indeed, these drugs are associated with long-lasting B-cell impairment, hypogammaglobulinemia, and need for immunoglobulin replacement. For these patients, inactivated vaccines should be delayed by at least 6 months after treatment, and live attenuated vaccines should be postponed for at least 12 months [19,20,21,22,23].

### 3.2. Vaccinating Children Receiving CAR-T Cell Therapy

Chimeric antigen receptor (CAR) T-cell therapy represents a groundbreaking advancement in the treatment of hematologic malignancies, especially those involving B lymphocytes (i.e., ALL). Patients with ALL who respond to anti-CD19 CAR T-cell therapy often develop B-cell aplasia, leading to hypogammaglobulinemia and increased infection risk [24,25,26]. Given the significant and durable B-cell impairment, vaccination plays a crucial role in reducing the burden of infections in these patients. However, data on the immunogenicity, efficacy, and safety of vaccines in this setting are scarce and there are no large-cohort data about the persistence of immunity after CAR-T cell therapy. Therefore, further research is needed to determine how well antibody-mediated immunity is preserved following CAR-T cell therapy and in optimizing revaccination timing and identifying hematologic markers that predict vaccine responses. Currently, recommendations are largely based on expert opinion [27]. As a general rule, non-live vaccination should be considered from 3 to 6 months after CAR-T cell infusion, while live vaccines can be administered at least 12 months after infusion. Apart from the timing, other factors should be considered before vaccinating CAR-T cell recipients. These parameters, which are also mentioned in current recommendations, include the absence of ongoing chemotherapy/HSCT, appropriate distance from the last immunoglobulin replacement therapy, and an adequate immune reconstitution (IR). For this population, IR is defined as CD4+ T cells > 0.2 × 10^9^/L and CD19+ or CD20+ B cells > 0.2 × 10^9^/L [28,29,30]. Additionally, it is important to consider that, in some cases, CAR-T cell therapy is used in patients with malignancy relapsing after HSCT. In this condition, vaccination schedules should also consider the interval from transplantation [28].

**Table 2:** Vaccination timing after treatment with monoclonal antibodies and CAR-T cell therapy in pediatric hematology and oncology (adapted from refs. [19,28]).

Treatment	Mechanism of Action	Non-Live Vaccines	Live Vaccines
Blinantunomab [19]	Anti-CD19	6 months	12 months
Inotuzumab-ozogamicina [19]	Anti-CD22	6 months	12 months
Rituximab (and other anti-CD20 therapies) [19]	Anti-CD20	6 months	12 months
Alemtuzumab [19]	Anti-CD52	6 months	12 months
Daratumomab [19]	Anti-CD38	17 weeks	17 weeks
Gentuzumab-ozogamicina [19]	Anti-CD33	5 weeks	12 weeks
Eculizumab [19]	Anti-C5	8 weeks	12 weeks
Brentuximab-vedotin [19]	Anti-CD30	4 weeks	12 weeks
CAR-T cell therapy [28]	Different targets (i.e., CD19 in ALL)	3 months (no ongoing immunosuppression, evidence of detectable IgA, at least 2 months from last IVIG)	12 months (no ongoing immunosuppression, evidence of immune reconstitution, detectable serum IgA, at least 8 months from last IVIG)

Despite potentially lower vaccine efficacy, consensus guidelines recommend IIV during flu season and pneumococcal conjugate vaccines due to the risk of invasive pneumococcal disease. HBV vaccination is also suggested, specifically in patients at high risk of infection or in regions with high prevalence. Vaccines should be repeated once B-cell aplasia has resolved. Furthermore, as the efficacy of vaccinations may be impaired in this population, it is strongly recommended to extend the immunization to family members and close contacts of patients undergoing CAR-T cell therapy [28,29,30].

## 4. Vaccinations in Children Receiving Hematopoietic Stem Cell Transplantation (HSCT)

Recipients of HSCT face a significantly increased risk of infection-related mortality, stemming from both opportunistic infections and vaccine-preventable diseases [31,32]. While IR is gradually achieved after transplantation, HSCT conditioning regimens result in the loss of immune memory to antigens encountered before the procedure. Consequently, HSCT recipients require revaccination, regardless of the original indication for transplantation. Despite numerous studies investigating the efficacy and safety of vaccinations in HSCT recipients, the optimal timing for vaccine administration remains uncertain. Additionally, the heterogeneity of HSCT procedures, including the increasing use of autologous HSCT for malignancies and its expanding indications (e.g., hemoglobinopathies, inborn errors of immunity [IEI], and metabolic diseases [33,34,35]), emphasizes the need for individualized vaccination strategies [36].

### 4.1. Kinetics of Immune Reconstitution: An Overview

Neutrophils are the first cell population to recover after HSCT, with the median time for neutrophil engraftment ranging from 14 to 20 days [37,38]. NK cell recovery begins early and is typically complete within the first month post-HSCT, generally observed within 4 months [37,38]. T-cell IR occurs in two distinct phases. The first phase is marked by the expansion of graft-derived memory T cells, while the second phase, starting around 6 months post-HSCT, is characterized by the thymic generation of naïve T cells derived from graft-provided lymphoid stem or progenitor cells [32]. CD8+ T cells tend to recover earlier than CD4+ T cells [39], and complete T-cell IR can take any time from 6 to 24 months, with a median of 12 months [39,40].

B-cell reconstitution depends on de novo generation of precursors in the bone marrow. Transitional B cells appear around 2 months post-HSCT [31,32,33,34,35,36,37,38,39,40,41], followed by a gradual increase in naïve B cells [11]. Memory B cells normalize later, around 24 months post-HSCT, with evidence of reduced non-switched memory B cells [41,42]. Immunoglobulin levels recover progressively: IgM in the first 3–6 months, IgG within the first year, and IgA as the last immunoglobulin to normalize the levels [41,42,43]. However, long-term deficiencies in immunoglobulin synthesis may persist, resulting in an impaired immune response to T-independent antigens. Moreover, HSCT recipients show a significant reduction in the diversity of the B-cell receptor (BCR) repertoire [44], which further compromises humoral immune responses. Notably, in clinical practice, the absence of a defined timing to study IR limits the harmonization of the literature data and, consequently, the knowledge about this topic.

### 4.2. Factors Influencing Immune Reconstitution

**Figure 1:**
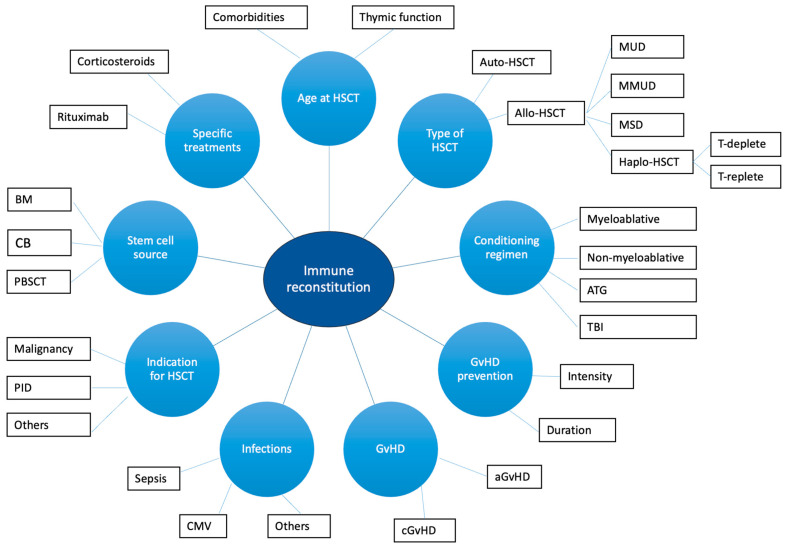
Factors affecting immune reconstitution in HSCT recipients. Figure legend: aGvHD: acute graft-versus-host disease; ATG: anti-tymocyte globulin; BM: bone marrow; CB: cord blood; aGvHD: acute graft-versus-host disease; CMV: citomegalovirus; MSD: matched sibling donor; MMUD: mismatched unrelated donor; MUD: matched unrelated donor; PBSCT: peripheral blood stem cell transplantation; TBI: total body irradiation.

IR is influenced by numerous factors, including graft-versus-host disease (GvHD), infections, age, type of HSCT (allogenic HSCT, autologous HSCT, haploidentical HSCT), the source of stem cells, the indication to HSCT, and conditioning regimen [37,38,45] (Figure 1). GvHD can affect IR through the induction of thymic damage, with consequent reduced thymic output of naïve T cells and reduced TCR repertoire diversity [46]. Moreover, corticosteroids administered to treat GvHD exacerbate apoptosis of thymocytes and thymic atrophy [46]. Infections (mainly, cytomegalovirus infection) can cause thymic damage [46]. Also, reduced levels of B-cell progenitors, non-switched memory B cells, and regulatory B cells are detected in patients with chronic GvHD (cGvHD) [41,46,47]. The decline in the thymic function observed with aging contributes to differences in the IR between adults and children [46]. The thymic function can also be impaired by myeloablative conditioning, cyclophosphamide, total body irradiation, and anti-thymocyte globulin [46,48]. On the other hand, rituximab negatively affects B-cell reconstitution and function [49]. Finally, patients undergoing HSCT for IEIs with altered thymic function show reduced naïve T cell recovery and reduced TCR repertoire diversity, as expected [50]. Data regarding the role of the stem cell source are still non-univocal [51]. However, according to the literature data, the use of cord-blood (CB) stem cells is associated with a rapid B-cell reconstitution compared to bone marrow (BM) and peripheral blood stem cell transplantation (PBSCT) [52,53,54]. On the other hand, patients receiving non-manipulated PBSCT commonly achieve a faster T-cell recovery [51]. Interestingly, in pediatric patients, long-term T-cell reconstitution develops with comparable timings between CB and BM recipients [55]. Finally, special considerations should be made regarding auto-HSCT and haploidentical HSCT.

Both in adults and children, patients undergoing auto-HSCT show a faster recovery of total lymphocytes, T helper and cytotoxic cells, higher thymopoiesis, and faster B-cell IR compared with allogenic HSCT [56]. Concerning haploidentical HSCT, IR is strongly influenced by the different transplantation platforms [57,58], which include strategies of ex vivo depletion of T cells [59] or nonselective lymphodeplete graft requiring post-HSCT immunosuppression [57,60].

### 4.3. Efficacy and Safety of Vaccinations Following HSCT

The response rate to the main vaccinations in pediatric and adult HSCT recipients has been widely investigated, as well as the safety profile in this population [61]. Data are summarized in Table 3. According to the literature data, non-live vaccines generally elicit a higher response compared to live attenuated vaccines [62,63,64,65,66,67,68,69,70,71,72,73,74,75,76,77,78,79,80,81,82,83,84,85,86,87,88,89,90,91,92,93,94]. The heterogeneity of clinical practice is reflected by the different timings reported in previous studies. Additionally, as a significant number of papers on this topic are dated, there is a need for updated studies. Although, as a general rule, a delayed revaccination schedule is associated with a higher response rate (also notably, with a higher early infection risk), studies on early vaccinations showed promising results for specific vaccines. A paradigmatic example is represented by pneumococcal vaccines. Comparing different studies, it emerges that the response ranges from 64% to 98%, and they do not show a significant variation between patients who started revaccinating after 3 or 9 months of HSCT, thus allowing the adoption of early vaccination schedules [67,68,69]. Differently, the response rate against influenza is significantly increased for patients undergoing vaccination after at least 6 months from HSCT [62,63,64,65,66,95]. Finally, anti-SARS-CoV-2 vaccines were widely administered at different times in HSCT recipients during the recent pandemic, thus showing relevant differences regarding the optimal timing of vaccination and number of required doses to achieve seroconversion [93,94]. Further studies on early revaccination after HSCT, specifically investigating also the role of ongoing treatments (i.e., cyclosporine, tacrolimus) are warranted. Indeed, given the high infectious risk in HSCT recipients, increasing knowledge about the efficacy of early revaccination strategies could significantly change current clinical practice.

The administration of non-live vaccines in HSCT recipients is not associated with relevant specific safety issues. On the other hand, the use of live vaccines is associated with concerns deriving from the risk of viral reactivation [88]. Regarding this, the use of delayed vaccination strategies and the avoidance of live vaccines in immunosuppressed patients, according to current guidelines [10,95,96,97], markedly reduced the risk of vaccine-related viral disease.

### 4.4. An Overview of Current Guidelines

The recommendation of the most recent 2017 European Conference on Infections in Leukaemia (ECIL7) guidelines, approved in 2019, and the 2023 ACIP guidelines are summarized in Table 4 and Table 5 [9,10,95,96]. Most of the current recommendations are directed to allogenic HSCT recipients only. Significant innovation was proposed in the most recent guidelines, which include specific indications for auto-HSCT and consider the occurrence of GvHD as a factor influencing the vaccination strategy [95]. According to available guidelines, HSCT recipients should be revaccinated independently from the presence of antigen-specific antibody titers, thus significantly limiting the utility of their assessment in clinical practice. Some exceptions to this rule are represented by live attenuated vaccines, where the presence of antigen-specific antibodies affects the vaccination strategy. As to non-live vaccinations, available guidelines agree on their administration 6 to 12 months post-HSCT [9,10,95,96,97,98,99], with some exceptions for the pneumococcal conjugated vaccine and inactivated polio vaccine, which can be administered 3 months post-HSCT. Also, the flu vaccine can be anticipated in case of an influenza outbreak.

The indications for attenuated vaccines are significantly more restrictive. Vaccination against mumps, measles, rubella, and varicella is possible 24 months post-HSCT in the absence of GvHD and immunosuppression in seronegative patients [9,10,95,96]. Notably, administration of the recombinant anti-zoster vaccine (RZV) is currently available only for the adult population following HSCT [90,91]. In the future, studies on RZV will hopefully define its applicability even in pediatric immunocompromised patients, including HSCT recipients.

Most of the other attenuated vaccines, including rotavirus and BCG, are not recommended or contraindicated [9,10,95,96]. However, the administration of yellow fever, tick-borne encephalitis, and rabies vaccines, can be considered in specific epidemiological situations [95].

## 5. Vaccinations in the Asplenic Children

The spleen is responsible for hemocatheresis and the regulation of innate and adaptive immune responses. A heterogeneous group of IgM memory B cells located in the marginal zone play a crucial role in mounting prompt responses against both viral and bacterial pathogens and in maintaining T cell-independent immune responses [100,101].

### 5.1. Asplenia and Hyposplenism: Clinical Implications

The term asplenia refers to the absence of the spleen, a condition that is rarely congenital and mostly acquired (i.e., post-surgical). Differently, the term hyposplenism refers to the acquired impairment of spleen functions [102]. The etiological causes of asplenia and hyposplenism are multiple and complex [103,104]. Hematologic disorders constitute the main indication for therapeutic splenectomy. This can be the case for hemoglobinopathies such as sickle cell anemia, red cell membrane anomalies (i.e., hereditary spherocytosis), and platelet disorders such as immune thrombocytopenic purpura (ITP) [105]. Some diseases are associated with a progressive risk of hyposplenism and include sickle cell disease (SCD), cGvHD, and a history of previous splenic irradiation [106]. Specifically, patients with SCD tend to develop functional asplenia in the first years of life, and anatomical asplenia due to multiple splenic infarctions before the age of 10 [107]. Since the first reported cases of fulminating sepsis among splenectomized children [108], it is now well-known that asplenia causes a higher susceptibility to bacterial sepsis. Following this, together with the development of new medical therapies, the indications for splenectomy for hematologic disorders were reduced [109]. The factors responsible for the increased incidence of severe infections following splenectomy include the insufficient opsonizing filter function of the spleen and delayed/impaired production of immunoglobulins [103,104]. Moreover, after splenectomy, an altered distribution of lymphocyte subpopulations, with a typical reduction in memory-switched B cells in the first 150 days post-splenectomy, [110,111,112,113] is observed. This causes a specifically increased risk for infections caused by polysaccharide-encapsulated bacteria, particularly Streptococcus pneumoniae [113], Neisseria meningitidis, and Haemophilus influenzae type B, together with a reduced response rate to polysaccharide vaccines. In some cases, asplenic patients can develop a severe and life-threatening condition called overwhelming post-splenectomy infection (OPSI), which has a very high mortality rate due to a rapid deterioration into full-blown fulminant septic shock within 24–48 h from the onset [114,115].

Although the highest risk for infections is during the first 3 years after surgical splenectomy, it remains elevated for life, as demonstrated by reports of late sepsis [113]. Before the introduction of specific vaccine recommendations, the reported incidence of sepsis was higher than 3% in patients receiving surgical splenectomy [107,116,117].

### 5.2. Vaccination Strategies in Patients with Asplenia

Despite adequate treatment, the mortality rate of OPSI remains high. The main prevention strategies include patient education, vaccination, and antibiotic prophylaxis [106].

Given the impairment in the T-cell independent immune response in asplenic patients, conjugate vaccines are preferred in the prevention strategy against encapsulated bacteria. Conjugate vaccines can induce the immune response independently of the antibody response by memory B cells, as their mechanism relies on the T cell-dependent pathway [118]. By contrast, polysaccharide vaccines rely on the T cell-independent pathway to initiate memory, which requires functioning memory B cells to develop an effective response [119,120]. Thus, the sequential use of conjugate and unconjugated vaccines is now generally recommended in patients with hyposplenism or asplenia to optimize the response [10,106,107]. Also, periodical booster vaccinations are needed to maintain long-term immunity. Current vaccine recommendations involve both the conjugate and unconjugated pneumococcal vaccines, quadrivalent conjugate meningococcal vaccine and recombinant meningococcal B vaccine, a conjugated H. influenzae type b vaccine, and the annual influenza vaccine [10,106,107] (Table 6).

Concerning surgical splenectomy, the timing of vaccination is pivotal when surgery is planned. In this case, vaccination is recommended at least 2 weeks before elective splenectomy. For patients undergoing emergency splenectomy, vaccines are recommended after at least 2 weeks from surgery, to ensure optimal antibody function and slower waning of antibody levels [107,121,122].

#### 5.2.1. Pneumococcal Vaccines

The sequence of administration is important for pneumococcal vaccines to optimize the immune response. As previously stated, conjugate pneumococcal vaccines such as PCV13 should be administered before polysaccharide vaccines (PPSV23). Indeed, previous studies reported that patients who receive PPSV23 as the initial dose have lower antibody responses, shorter duration of immunity, and hyporesponsiveness to subsequent doses of either vaccine [121,122]. As summarized in Table 6, the number of doses of PCV13 varies according to the patient’s age and the previous immunization schedule. PPV23 doses should be limited to a total of two lifetime doses to avoid immune tolerance, which is commonly seen with repeated polysaccharide vaccination [123,124,125,126]. Although current data on sequential vaccination with either PCV15 or PCV20 followed by PPV23 are sparse, general guidelines are likely to change to schedules using the higher valent vaccines [127].

#### 5.2.2. Meningococcal Vaccines

For the prevention of invasive meningococcal diseases, patients with hyposplenism or asplenia should receive both the quadrivalent (ACWY) meningococcal and the meningococcal B vaccine [10,106,107,128,129]. MenACWY-CRM can be administered to children as young as 8 weeks of age, and the dosing schedule varies according to the age of the initial vaccination. A booster dose is recommended for those less than seven years old three years after primary vaccination and every five years afterward. The meningococcal B vaccine should be given according to the age-specific recommendation in the product information. Despite the lack of data, immunological considerations suggest that booster vaccinations should be administered every 5 years [130]. In the following years, the results of studies on the duration of anti-meningococcal B response will probably lead to an update of current recommendations. Specifically, the number of doses to administer in immunocompromised patients and the timing of booster doses represent two of the most relevant areas for further update.

#### 5.2.3. Haemophilus Influenzae Type B Vaccines

As the mortality for H. influenzae-associated OPSI is elevated [131], the Hib vaccination continues to be strongly recommended in previously non-vaccinated people with asplenia. Due to the excellent control of HiB depending on long-standing successful vaccination programs in most countries, recent guidelines agree that additional HiB vaccination is no longer recommended for fully vaccinated children even after surgical splenectomy [10,106,107].

## 6. Future Directions: Towards a Personalized Vaccine Strategy in Pediatric Hematology and Oncology

The challenge of personalized vaccine strategy is of significant clinical relevance in the pediatric hematology and oncology setting. Indeed, current guidelines highlight some universal parameters (i.e., timepoints from the end of chemotherapy or HSCT) to define the vaccine schedule. However, there is substantial heterogeneity among patients in these categories. To address this variability, vaccine strategies tailored to each patient’s clinical and immunological profile are essential, with the final aim of reducing the infectious risk in this delicate category of patients.

### 6.1. Role of the Immunological Assessment to Evaluate IR After Chemotherapy

Few studies explored the dynamics of immune reconstitution following chemotherapy in children with hematological and oncological disorders. Furthermore, different factors might affect immune recovery, including background, type of malignancy, intensity of chemotherapy, use of B-cell targeting antibodies, and age [132]. In addition, an extensive characterization of lymphocyte subpopulation (i.e., naïve/memory cell subsets) recovery following the cessation of treatment in children is lacking. According to available data, within B cell subsets, naïve B cells appear to be more affected, with a lower relative proportion as compared to memory B cell subsets. The kinetic of B-cell recovery is widely variable. Indeed, it may begin as early as one month post-treatment, but full recovery can take up to 18 months, especially in children treated with anti-CD20 monoclonal therapies [133,134,135]. Notably, protective antibodies acquired through primary vaccinations are often lost during treatment, leaving children vulnerable to vaccine-preventable diseases [136]. All this considered, immune recovery assessment is of paramount importance in designing effective strategies for reimmunization. Although it is not part of current clinical practice, the analysis of lymphocyte subpopulations and, when available, extended lymphocyte phenotyping, could potentially help in identifying the optimal timing to administer vaccines after the end of chemotherapy.

As previously discussed, the majority of guidelines suggest considering re-vaccination 3–6 months after chemotherapy cessation [137]. However, this represents a common historical practice rather than being guided by immune status after anti-neoplastic treatment. Concerning this, the rapid recovery in naïve B cells observed in patients after the end of chemotherapy can provide the rationale for very early immunization. This is supported by a recent study by Bate et al. demonstrating that the end of chemotherapy represents the earliest timepoint in which protective anti-pneumococcal immunity can be achieved [138]. This study compared the rate of vaccine response in patients with ALL who received anti-pneumococcal vaccination during the maintenance phase, at the end of chemotherapy, or 6 months after the end of chemotherapy. Patients vaccinated at the end of chemotherapy achieved a similar response rate (59.5% vs. 56.8%) and persistence of response after 12 months (37.9% vs. 43.3%) compared to patients receiving immunization after 6 months. Notably, this study is limited by the small number of patients and the lack of an immunological assessment in responders and non-responders. However, it opens intriguing discussions on early revaccination strategies, especially in patients carrying risk factors for invasive pneumococcal disease. This reinforces the need for an accurate and periodical immunological assessment, to finally provide a tailored vaccination strategy.

### 6.2. From Time-Based to Immunology-Based Vaccination Schedule in HSCT Recipients

Given the heterogeneity of HSCT indications, conditioning regimens, and the wide range of factors influencing IR, the vaccination strategy in HSCT recipients represents a considerable challenge. Moreover, there is no universal definition of IR, and different cut-offs of CD3+, CD4+, and CD19+ cells were used in previous studies.

To provide a more personalized vaccine approach, recent studies attempted to identify laboratory thresholds for guiding revaccination, including minimum ALC, serum immunoglobulin levels, and absolute CD4+ and CD19+ counts [139,140,141]. For instance, Haynes et al. recently reviewed an immune recovery-based protocol that prescribes a minimum time interval and immunologic benchmarks before vaccination [139]. In this study, the authors considered minimum laboratory criteria, which included an ALC > 1000/µL, CD4+ > 400/µL, and IgG > 400 mg/dL. These laboratory criteria were integrated with clinical parameters including time from HSCT, ongoing therapies, GvHD, and the interval from the last immunoglobulin administration. This approach allowed for the observation of higher rates of seroprotection after vaccination using an IR-based protocol. Despite this promising result, evidence is currently limited, and standardized IR-based schedules are currently unavailable in clinical practice. This depends on the lack of evidence-based lymphocyte and immunoglobulin thresholds deriving from larger cohorts. Therefore, specific studies on this topic are warranted.

Beyond the baseline immunological assessment, the study of thymic and bone marrow output, together with the characterization of the TCR and BCR repertoire, diversity could significantly improve the understanding of IR. The evaluation of thymic output mostly relies on the analysis of extended lymphocyte phenotyping, with a specific focus on recent thymic emigrants (RTE) [142]. More recently, the possibility of quantifying T-receptor excision circles (TRECs) using real-time PCR techniques gave new interesting insights into the analysis of thymic output [143]. The evaluation of bone marrow output, with a focus on the crucial stages of B cell reconstitution, can be studied at a similar level, through the quantification of K-receptor excision circles (KRECS). KRECS can be detected after a variable timing from HSCT, ranging from 2 to 6 months, and express the initial recovery of transitional B cells [144]. Some studies investigated the potential correlations between TRECS and KRECS levels and HSCT outcome, including the occurrence of infections, viral reactivation, and the prediction of GvHD and relapse of the underlying disease. However, definitive results and specific cut-offs are still lacking [145,146,147,148,149].

**Figure 2:**
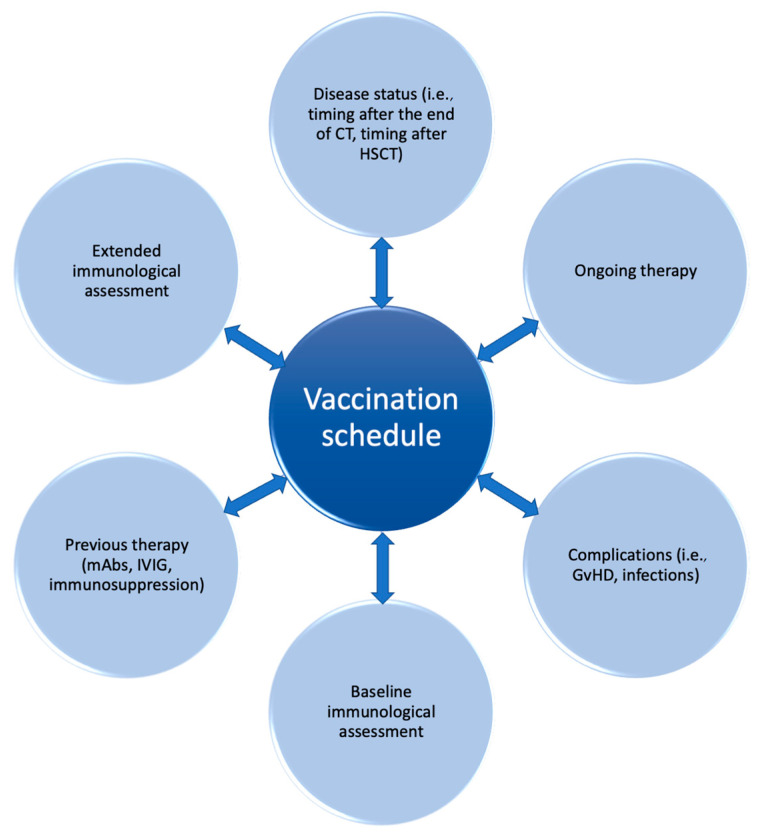
Vaccination strategy in pediatric hematology and oncology: a personalized perspective.

The ability of the immune system to respond to foreign antigens, including vaccines, derives also from the diversity of the BCR and TCR repertoire, which can be significantly reduced in HSCT recipients. The analysis of the TCR and BCR repertoire can be performed with different techniques, including Southern blot, flow cytometry, RT-PCR, and new sequencing methods [150]. It is well known that HSCT recipients show reduced repertoire diversity, which progressively increases with IR [41,151]. The study of thymic and bone marrow output and T-cell and B-cell repertoire was specifically investigated in correlation with vaccine strategies. This analysis, although currently limited in clinical practice, represents an intriguing perspective from which to expand the knowledge about functional IR and a potential outcome predictor, and could pave the way to tailored vaccine schedules in terms of optimal vaccine response rate and safety for the patient (Figure 2).

## 7. Conclusions

Vaccination in children receiving chemotherapy, HSCT, or those affected by asplenia, represents a unique challenge due to immunosuppression and the variable timeline of immune recovery. Adherence to current guidelines is crucial in optimizing the timing and selection of vaccines in specific populations, minimizing the risk of infections, and improving long-term outcomes for pediatric hemato-oncologic patients. Ultimately, implementing a personalized approach that considers individual patient factors such as the underlying disease, treatments received and immune status, is critical to ensuring effective and safe vaccination strategies. Advances in specific research areas will hopefully lead to further improvement in the management of this vulnerable population. These include the definition of specific timepoints for the assessment of IR after chemotherapy and HSCT, the identification of lymphocyte and immunoglobulin thresholds to start revaccination, and the implementation of thymic and bone marrow output assessments in clinical practice.

## Figures and Tables

**Table 1:** Vaccinations in children during and after chemotherapy.

Vaccine	During Chemotherapy	After Chemotherapy
Inactivated influenza	Recommended annually.	Recommended annually.
Conjugated pneumococcal vaccine	Recommended at diagnosis of malignancy.	Possible administration 3 months after the completion of chemotherapy.
COVID-19	Recommended during pandemic.	Recommended as for the general population.
HBV	Consider vaccination of seronegative patients before starting/during chemotherapy (in highly endemic areas).	Possible administration 3 months after the completion of chemotherapy.
Other inactivated vaccines [9,10]	Non contraindicated during maintenance phase (consider non-valid doses unless a protective response is demonstrated).	Possible administration 3 months after the completion of chemotherapy.
Varicella [5,10]	Contraindicated.	From 3 to 12 months after the completion of chemotherapy (at least 6 months if anti-B-cell therapies have been used).
MMR [5,10]	Contraindicated In case of measles epidemic, it is possible to administer measles vaccine in patients with adequate CD4+ levels (>500/μL if >12 months) [12].	From 3 to 12 months after the completion of chemotherapy (at least 6 months if anti-B-cell therapies have been used)

**Table 3:** Vaccine Efficacy after HSCT.

	Timing After HSCT and Schedule	Response Rate
Influenza [31,32,33,34,35,36,65]	<6 months >6 months	10–40% 10–84%
Conjugated Pneumococcal [61,67,68,69]	3–9 months	64–98%
Tetanus [61,71,73,74,75]	6–12 months (three doses)	85–100%
Pertussis [61,73,79,95]	12–17 months	25–54%
Dyptheria [71,75,80]	3–18 months	70–100%
Haemophilus influenzae B [75,76,81,82]	3–12 months	80–100%
Polio [71,73,75,76,77,78]	6 months (three doses)	80–100%
HBV [70,71,72]	Median: 23 months (three doses)	64–82%
Meningococcus ACWY [83,84,95]	12–18 months	76–100%
Meningococcus B	No available data	
HPV [85]	study on a wide range of immunocompromised children	88–100%
HAV [86]	11 months (median)	33%
SARS-CoV-2 mRNA vaccines [93,94]	Variable	38–81%
Varicella [61,87,88,89]	24 months (median)	58–80%
Inactivated varicella zoster [90,91]	5–50 days (first dose)	64–76%
Measles [73,76,89,92,95]	24 months	65–100%
Mumps [73,76,89,92,95]	24 months	50–87%
Rubella [73,76,89,92,95]	24 months	75–100%

**Table 4:** Main guidelines on non-live vaccines in HSCT recipients.

	ACIP 2023 [9,10,96]	ECIL7 2019 [95]
Inactivated influenza vaccine	Recommended from 6 months from HSCT (consider after 4 months in case of epidemic). Consider a second dose in children receiving the first vaccination < 9 years. Consider starting vaccination 4 months after HSCT (in this case, a second dose should be considered).	Recommended from 6 months from HSCT (consider after 3 months in case of outbreak). Consider a second dose after 4 weeks in children < 8 years, severe GvHD, lymphopenia.
Pneumococcus	From 3 to 6 months after HSCT; recommended four doses of PCV-20 (the first three should be separated by 4 weeks; 6 months for the third and fourth). Alternative: three doses of PCV15, followed by a dose of 23 V at 12 months from the third dose. In patients with GvHD, administer a fourth dose of PCV15 instead of PPSV-23.	From 3 months after HSCT, recommended three doses of 13 V with 1 month interval. If severe GvHD: fourth dose of 13 V at least 6 months from the third dose. 12 months after HSCT: a dose of 23 V is recommended (at least 8 weeks after the last 13 V) in patients receiving allogenic HSCT who do not have chronic GvHD.
HBV	From 6 months from HSCT: three doses. If postvaccination anti-HBs concentration of ≥10 mIU/mL not achieved: consider a second three-dose series [10].	For HBV negative patients: 6–12 months after HSCT, three doses 0, 1, and 6 months apart. Children should receive a standard pediatric dose (10 μg) of vaccine and adolescents should receive 20 μg dose.
Tetanus	Three doses from 6 to 12 months (see indications for pertussis-containing vaccines).	From 6 months from HSCT: three doses distanced at least 1–2 months.
Polio P IPV	6–12 months from HSCT: three doses of IPV.	IPV: three doses from 6 to 12 months after HSCT.
Pertussis	Indications for pertussis-containing vaccines: From 6 months from HSCT: three doses of DTaP for children aged <7 years. For patients ≥7 years, three options: (a) three doses of DTaP; (b) one dose of Tdap and two doses of DT; (c) one dose of Tdap and two doses of Td [10].	From 6 months from HSCT: three doses distanced by at least 1–2 months (use vaccine with higher dose of pertussis toxoid).
Dyptheria	Three doses from 6 to 12 months (see indications for pertussis-containing vaccines).	From 6 months from HSCT: three doses distanced at least 1–2 months.
Haemophilus	From 6 months after HSCT: three doses of anti-HIB (at least 1 month of interval).	From 3 months from HSCT: three doses distanced at least 1–2 months. Alternative: administration of three doses of a combined tetanus-dyptheria-pertussis-Hib vaccine from 6 months after HSCT.
Meningococcus	MenC: 6–12 months after HSCT, two doses of tetravalent vaccine for person aged 11–18 years. For those starting vaccinations at 11–15 years, administer a booster dose at 16–18 years [10].	MenC: From 6 months, two doses of monovalent or tetravalent vaccine. MenB: From 6 months, two doses.
HPV	From 6 to 12 months after HSCT, three doses in women aged 11–26 years (consider also in males) [10].	From 6 months after HSCT, following national recommendations.

**Table 5:** Main guidelines on live vaccines in HSCT recipients.

	IDSA 2013	ECIL7 2019
MMR	Two doses administered at least 24 months after HSCT, in seronegative patients without active GvHD, recent (<8–11 months) IVIG administration, or ongoing immunosuppression.	At least 24 months after HSCT, in patients without active GvHD, disease relapse, or ongoing immunosuppression and who are seronegative for antibodies against measles or (for women) rubella. In children, consider two doses 4 weeks apart. In case of measles epidemic, consider the administration 12 months after HSCT.
Varicella	Two doses administered at least 24 months after HSCT, in seronegative patients without active GvHD, recent (<8–11 months) IVIG administration, or ongoing immunosuppression and who are seronegative for varicella IgG antibodies.	At least 24 months after HSCT, in patients without active GvHD, disease relapse, or ongoing immunosuppression and who are seronegative for varicella IgG antibodies. In children, consider two doses 4 weeks apart.
Rotavirus	Not recommended	Not recommended
BCG	Not recommended	Not recommended
Yellow fever	Decision depending on individual risk assessment	At least 24 months after HSCT, in patients without active GvHD, disease relapse, or ongoing immunosuppression. Consider only if patients cannot avoid traveling to endemic areas.

**Table 6:** Vaccinations in patients with anatomical or functional asplenia/hyposplenia [10,106,107].

Vaccine	Before Elective Splenectomy	After Splenectomy and Other Causes of Asplenia/Hyposplenia
Conjugated pneumococcal	One dose at least 2 weeks before splenectomy if not previously vaccinated.	Age < 2 years: four doses of PCV13 (8 weeks apart) according to recommendations for the general population. Age 2–5 years: one additional dose of PCV-13 if previously vaccinated with three doses of conjugate vaccine; two doses of PCV-13 if not previously vaccinated. Age > 6: At least one dose of PCV-13.
Polysaccharide pneumococcal	One dose 8 weeks after PCV13. At least 2 weeks before splenectomy.	One dose of PPSV23 8 weeks after the last PCV13. Second dose of PPSV23 after 5 years.
Meningococcal C vaccines	Two-dose series of MenACWY, with the second dose given after 12 months of age (at least 8–12 weeks from first dose). The schedule should be completed at least 2 weeks before splenectomy. Booster dose every 5 years (if the most recent dose was administered before the age of 7, consider a first booster after 3 years, with following doses every 5 years).	Two-dose series of MenACWY, with the second dose given after 12 months of age (at least 8–12 weeks from first dose). Booster dose every 5 years (if the most recent dose was administered before the age of 7, consider a first booster after 3 years, with following doses every 5 years).
Meningococcal B vaccines	Vaccination according to recommendations for the general population. The schedule should be completed at least 2 weeks before splenectomy.	Vaccination according to recommendations for the general population. Start at lest 2 weeks after splenectomy.
Haemophilus influenzae B	Only in previously non vaccinated patients: one dose of HiB conjugate vaccine at least 2 weeks before splenectomy.	Follow recommendations for the general population. In previously non vaccinated patients who undergo splenectomy: one dose of HiB conjugate at least 2 weeks after splenectomy.
Influenza vaccine	Recommended annual vaccination.	Recommended annual vaccination.

## Data Availability

Not applicable.

## Abbreviations

The following abbreviations are used in this manuscript:

AIEOP	Italian Association for Pediatric Hematology Oncology
ALC	Absolute lymphocyte count
ALL	Acute lymphoblastic leukemia
BCR	B-cell receptor
BM	Bone marrow
CB	Cord blood
CDC	Centers for Disease Control and Prevention
CAR-T	Chimeric antigen receptor-T cell
cGvHD	Chronic graft-versus-host disease
ECIL7	2017 European Conference on Infections in Leukaemia
GvHD	Graft-versus-host disease
HBV	Hepatitis B
Hib	Haemophilus influenza type b
HSCT	Hematopoietic stem cell transplantation
IDSA	Infectious Diseases Society of America
IEI	Inborn errors of immunity
IR	Immune reconstitution
ITP	Immune thrombocytopenic purpura
IVIG	Intravenous immunoglobulin
KRECs	K-receptor excision circles
mAbs	Monoclonal antibodies
NK	Natural killer
OPSI	Overwhelming post-splenectomy infection
PBSCT	Peripheral blood stem cell transplantation
RTE	Recent thymic emigrants
SCD	Sickle cell disease
TCR	T-cell receptor
TRECs	T-receptor excision circles
